# RNA:protein ratio of the unicellular organism as a characteristic of phosphorous and nitrogen stoichiometry and of the cellular requirement of ribosomes for protein synthesis

**DOI:** 10.1186/1741-7007-4-30

**Published:** 2006-09-05

**Authors:** Tatiana V Karpinets, Duncan J Greenwood, Carl E Sams, John T Ammons

**Affiliations:** 1 Department of Plant Sciences, University of Tennessee, 2431 Joe Johnson Drive. Knoxville, TN 37996-4561, USA; 2 Warwick HRI, Warwick University, Wellesbourne, Warwick, CV35 9EF, UK; 3 Biosystems Engineering & Environmental Science Department, University of Tennessee, 2506 E. J. Chapman Drive, Knoxville, TN 37996-4531, USA; 4 Computer Science and Mathematics Division, Oak Ridge National Laboratory, P.O. Box 2008, MS6164, Oak Ridge, TN 37831, USA

## Abstract

**Background:**

Mean phosphorous:nitrogen (P:N) ratios and relationships of P:N ratios with the growth rate of organisms indicate a surprising similarity among and within microbial species, plants, and insect herbivores. To reveal the cellular mechanisms underling this similarity, the macromolecular composition of seven microorganisms and the effect of specific growth rate (SGR) on RNA:protein ratio, the number of ribosomes, and peptide elongation rate (PER) were analyzed under different conditions of exponential growth.

**Results:**

It was found that P:N ratios calculated from RNA and protein contents in these particular organisms were in the same range as the mean ratios reported for diverse organisms and had similar positive relationships with growth rate, consistent with the growth-rate hypothesis. The efficiency of protein synthesis in microorganisms is estimated as the number of active ribosomes required for the incorporation of one amino acid into the synthesized protein. This parameter is calculated as the SGR:PER ratio. Experimental and theoretical evidence indicated that the requirement of ribosomes for protein synthesis is proportional to the RNA:protein ratio. The constant of proportionality had the same values for all organisms, and was derived mechanistically from the characteristics of the protein-synthesis machinery of the cell (the number of nucleotides per ribosome, the average masses of nucleotides and amino acids, the fraction of ribosomal RNA in the total RNA, and the fraction of active ribosomes). Impairment of the growth conditions decreased the RNA:protein ratio and increased the overall efficiency of protein synthesis in the microorganisms.

**Conclusion:**

Our results suggest that the decrease in RNA:protein and estimated P:N ratios with decrease in the growth rate of the microorganism is a consequence of an increased overall efficiency of protein synthesis in the cell resulting from activation of the general stress response and increased transcription of cellular maintenance genes at the expense of growth related genes. The strong link between P:N stoichiometry, RNA:protein ratio, ribosomal requirement for protein synthesis, and growth rate of microorganisms indicated by the study could be used to characterize the N and P economy of complex ecosystems such as soils and the oceans.

## Background

Although microorganisms are flexible in their nitrogen (N) and phosphorus (P) content because of their ability to store these nutrients, the mean P:N ratios and their variations indicate surprising similarity among and within microbial species and even with plants and insect herbivores. For example, for marine phytoplankton, the variations in P:N ratio by mass are between 0.04 and 0.30, with an overall average close to the Redfield ratio of 0.14 [1]. An average value of the ratio in terrestrial plants at their natural field sites is in the range 0.07–0.08 [2-4]. Freshwater zooplankton and insect herbivores from terrestrial ecosystems also show similarity, with mean P:N ratios of 0.10 and 0.08 respectively [2].

Stoichiometric constraints are an important factor in the regulation of microbial growth and in their interaction with the environment. It has been proposed that an increase in the growth rate requires an increase in protein biosynthesis and, therefore an additional allocation of cellular resources to the synthesis of ribosomes and ribosomal RNA, which comprise the main part of cellular RNA. As a result of this allocation, the P:N ratio in the cell increases. This idea is referred to as the growth-rate hypothesis (GRH) [5-7]. In agreement with this hypothesis, the synthesis of rRNA in bacteria is maintained in proportion to the cell's requirement, and the number of rRNA genes correlate with the rate at which phylogenetically diverse bacteria respond to resource availability [8,9]. In marine bacteria, species-specific growth rates may be estimated by measuring the rRNA content of the organisms [10].

Ecological studies supporting the GRH usually consider P:N stoichiometry, growth rate, and RNA content across different organisms. A significant effect of the growth rate of an individual unicellular organism on its macromolecular composition has also been well-documented [11-16]. It was found that an impairment of growth in bacteria, yeast, algae, and fungi resulted in a decrease in their total RNA, rRNA, protein contents, and RNA:protein ratio. A direct proportionality between specific growth rate and RNA:protein ratio was reported in *Escherichia coli *[11]. This finding is in agreement with the GRH, because N and P in RNA and proteins dominate the total amounts of these elements in cells of unicellular organisms [5]. We can conclude from these studies that some molecular mechanisms activated in the cells of an individual organism in response to environmental conditions may be responsible for changes in P:N stoichiometry. At present, however, these mechanisms are not clear. According to studies of translational efficiency *in vitro *[17,18], the most rapid bacterial growth demands maximally efficient ribosomes. Because the number of ribosomes is proportional to cellular rRNA and RNA contents [19], the increased efficiency of protein synthesis by ribosomes under the faster growth reported in these studies may lead to a decrease in the RNA:protein ratio in the cell. However, this view is not in agreement with the GRH and is not supported by the aforementioned studies of macromolecular composition. In addition, studies of ribosomal, messenger, and transfer RNA in yeast during a nutritional shift [20] show that, although ribosomal RNA is significantly reduced after growth inhibition, messenger and transfer RNA are slightly increased [21]. These observations give indirect support to the notion that synthesis of proteins in microorganisms under growth impairment may require fewer ribosomes than under favorable growth, leading to a decrease in RNA:protein and P:N ratios. This cellular regularity may underlie the effect of growth rate on RNA:protein and P:N ratios at the level of an individual organism. The objective of this study was to investigate this hypothesis and to show that the cellular P:N ratio estimated from the RNA:protein ratio reproduces mean P:N ratios obtained experimentally for diverse microorganisms and plants. We analyzed macromolecular composition, number of active ribosomes, and peptide elongation rate in a set of unicellular prokaryotic and eukaryotic organisms (bacteria, yeast, fungi, algae) as functions of their growth rates. This information was used to calculate P:N stoichiometry of unicellular organisms and the cellular requirement of ribosomes for protein synthesis in different growth conditions.

It was found that the estimated P:N ratios derived from the RNA and protein contents of these organisms were in the same range as mean ratios reported for other, diverse organisms. There was a direct proportionality between the ribosomal requirement for protein production in the cell and RNA:protein ratio, which may underlie this similarity and the positive relationship of P:N ratio with the growth rate of the microorganisms. A decrease in the ribosomal requirement for protein production with an increase in the growth rate could be explained in terms of the disposal soma theory.

## Results

### P:N ratios calculated from RNA and protein contents of the unicellular organisms are in the same range as found in the primary producers and have similar relationships with growth rate

Experimental data for the analysis were obtained from previous studies of macromolecular composition (protein and RNA contents) of bacteria (*E. coli, Streptomyces coelicolor, Mycobacterium bovis, Selenomonas ruminantium*), budding yeast (*Saccharomyces cerevisiae*; two studies), fungi (*Neurospora crassa*) and algae (*Prototheca zopfii *) (Table 1). The studies are referred to by the name of the organism.

The masses of RNA and proteins in the studies were determined under different conditions of exponential growth characterized by a range of specific growth rates (SGR). This parameter is a measure of the number of divisions per cell per unit time. Between 1 and 12 growth conditions were considered in each study (Table 1), with SGR varying from 0.029 to 1.73 per hour. The variation was created by the use of different growth media. The P:N ratios calculated from RNA and protein contents in the studied organisms growing in such diverse conditions were in the range 0.04–0.16, with an average value of 0.1 (Table 1). This range of values was similar to that of the mean P:N ratios of the different organisms discussed in the introduction. The different growth conditions analysed in the study resulted in changes in P:N ratios in the microorganisms by factors of 5 and 4 only, whereas the range of each nutrient varied by three orders of magnitude (Table 1, columns 5–10). Smaller changes in P:N ratio at the organism level may be demonstrated by expressing the growth rate of the organisms as the relative growth rate (RGR). This parameter is often used in ecological studies [7], and is calculated as

**Figure 1:**
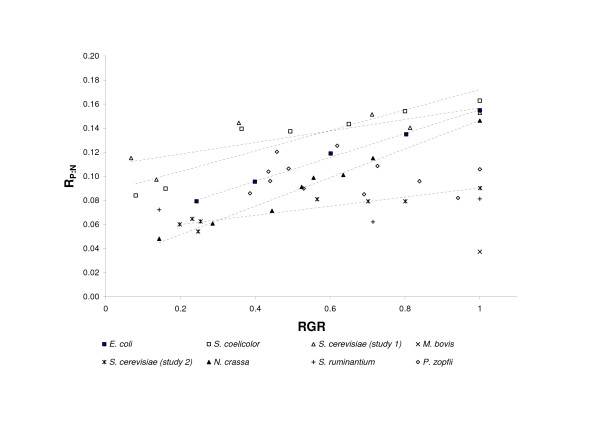
**The effect of relative growth rate on P:N ratio inthe organisms. **Relationships for *P. zopfii *and *S. ruminantium *are not presented by lines because of low correlation between the ratios and growth rates.

RGR = SGR/SGR_max_,

where SGR_max _is the maximum SGR of the organism in the study. Figure 1 and Table 2 illustrate the effect of RGR on P:N ratio.

**Figure 2:**
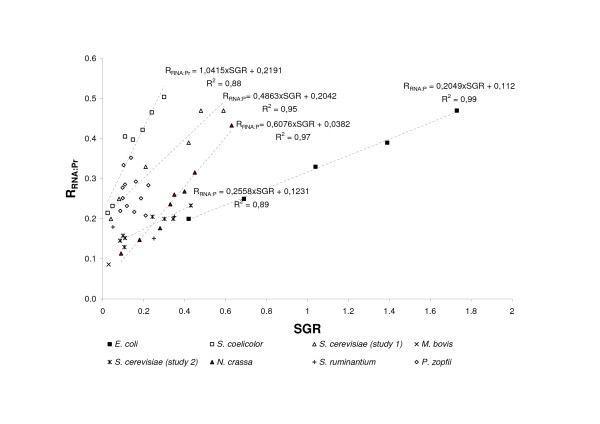
**The effect of specific growth rate on RNA:protein ratio in the organisms. **Relationships for *P. zopfi*i and *S. ruminantium* are not presented by lines because of low correlation between the ratio and specific growth rate.

In all organisms, except the algae *P. zopfii *and *S. ruminantium *,an increase in growth was accompanied by a linear increase in RNA:protein ratio (Figure 2) and P:N ratio (Figure 1). The ratios in *P. zopfii *showed no correlation with growth (Table 2), possibly because the RNA:protein ratio varied only within the narrow range 0.22–0.28, which corresponds to 0.09–0.13 for the P:N ratio. The study of *S. ruminantium *included only three growth conditions, and, similarly to *P. zopfii *,the P:N ratio of the organism varied only slightly (from 0.072 to 0.081). In agreement with the GRH, the P:N ratio calculated from RNA and protein contents increased with an increase in the specific growth rate of the unicellular organism. The rate of increase in P:N ratio per unit of RGR, which was estimated as the slope of the regression line (Table 2), varied among the unicellular organisms, from 0.06 in *E. coli *to 0.28 in *S. coelicolor*. The intercept of the regression line on the ordinate also varied among organisms, even between different strains of *S. cerevisiae*. Considering the strong correlations between P:N ratios and RGR for most organisms, demonstrated by Figure 1, the regression intercepts may characterize the P:N ratios required to maintain organisms in the stationary state.

### The cellular requirement of ribosomes for protein production in microorganisms may be estimated by the RNA:protein ratio using a common constant characterising protein synthesis machinery in the cell

The mass of ribosomal RNA per cell (m_rRNA_) measured in the studies of *E. coli, S. coelicolor, M. bovis, S. cerevisiae *(two studies), and *N. crassa *(Table 3) allowed us to estimate the cellular requirement of ribosomes for protein synthesis in the organisms as functions of the growth conditions by calculating the number of ribosomes and the peptide elongation rate (PER). By definition, PER is the number of amino acids incorporated into the peptide chain per second by one active ribosome. Therefore, it was calculated as the rate of protein synthesis in the cell expressed in amino acids per second divided by the number of active ribosomes, or algebraically as

(1)     PER = n_aa _× SGR/(F_Ra_× n_R_),

where F_Ra _is the fraction of active ribosomes (involved in protein synthesis), n_aa _is the cellular protein content expressed in amino-acid equivalents, SGR is the specific growth rate expressed per second, and n_R _is the number of ribosomes per cell.

The number of ribosomes per cell is estimated from m_RNA _by the equation

(2)     n_R _= F_rRNA_× m_RNA_× N_a_/(M_nucl _× n_nuclR_),

where F_rRNA _is the fraction of ribosomal RNA in m_RNA _calculated as the ratio m_rRNA _and m_RNA_, N_a _is Avogadro's constant, M_nucl _is the average mass of the nucleotide, and n_nuclR _is the number of nucleotides per ribosome.

To characterize the cellular requirement of ribosomes for protein synthesis under different growth conditions, we calculated the increase in SGR per unit increase in PER, i.e. the ratio SGR:PER (R_SGR:PER_). From equation 1, the following expression for this ratio was obtained:

(3)     R_SGR:PER _= (F_Ra_× n_R_)/n_aa_

**Figure 3:**
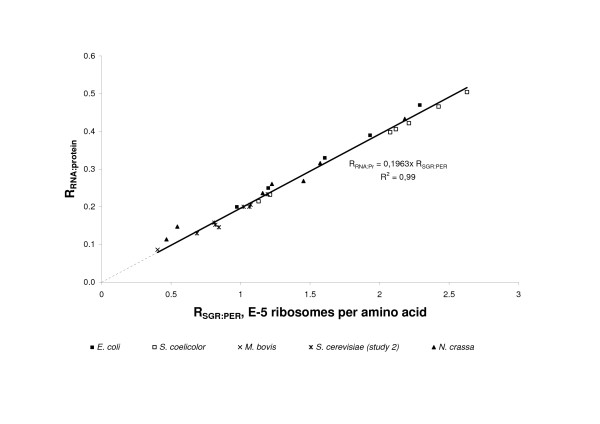
**The proportional relationship between the RNA:protein ratio and the number of active ribosomes per synthesized proteins. **The slope of the line is equal to the C value in equations 6–8. The mass of ribosomal RNA was not measured in studies of *S. ruminantium *and *S. cerevisiae *(one study), therefore only five species are considered.

According to this equation, R_SGR:PER _measures the number of ribosomes in the cell required to incorporate one amino acid into a new protein. The reciprocal of this parameter (PER:SGR) is a measure of how efficiently synthesized ribosomes are used in the cell for protein production. In addition to the intrinsic translational efficiency of ribosomes, which may be estimated by studies of translation *in vitro *[22,23], this parameter also includes the effects of cellular environment on the stability of mRNA, proteins and ribosomes. Therefore, the SGR:PER ratio gives an indirect estimate of the overall efficiency of cellular protein synthesis *in vivo*. It makes the parameter more suitable for estimating the effect of growth conditions on the efficiency of protein-production machinery of the microorganism. Values of R_SGR:PER _in the microorganisms calculated in the studies are summarized in Table 3. Figure 3 presents experimental evidence for a close correlation (*R*^2 ^= 0.98) between the values of R_SGR:PER_, which characterizes the ribosomal requirement for protein production, and the RNA:protein ratio (R_RNA:Pr_). Moreover, this relationship has the same coefficient of proportionality for all organisms and growth conditions, referred to below as the C value.

Next we considered which cellular parameters are responsible for the identified proportional relationship between R_SGR:PER _and the RNA:protein ratio. Substitution of equation 2 into equation 3 gives

(4)     R_SGR:PER _= F_Ra_× F_rRNA_× m_RNA_× N_a_/(M_nucl_× n_nuclR_× n_aa_)

Considering n_aa_= m_Pr_× N_a_/M_aa_, where M_aa _is the average mass of the amino acid, the equation underlying the proportional relationship between R_RNA:Pr _and R_SGR:PER _demonstrated in Figure 3 may be expressed as

(5)     m_RNA_/m_Pr _= (M_nucl_× n_nuclR_)/(F_Ra_× F_rRNA_× M_aa_) × R_SGR:PER_

or

(6)     R_RNA:Pr_= C × R_SGR:PER,_

where

(7)     C = (M_nucl_× n_nuclR_)/(F_Ra_× F_rRNA _× M_aa_).

If the left-hand masses of RNA and proteins in equation 5 are expressed in nucleotide and amino-acid equivalents, respectively, then

(8)     C = n_nuclR_/(F_Ra_× F_rRNA_).

Equations 7 and 8 represent an analytical expression for the C value found in the experimental observations and demonstrated by Figure 3. Separate quantification of each parameter in equation 7 helps explain the characterization of the ribosomal requirement for protein production by the RNA:protein ratio for different microorganisms and growing conditions.

• M_nucl _and M_aa_. The average masses of nucleotide and amino acid in equation 7 are about 330 and 120 Da respectively and are about the same in different organisms.

• n_nuclR_. The transcripts of ribosomal subunits also have high sequence similarity. The number of nucleotides comprising the ribosome, parameter n_nuclR_, is rather conservative across a wide range of species. Only small differences in the size of rRNA in different bacteria have been reported [24]. The 16S rRNA varies between 1487 bp for the cyanobacterium *Anacystics nidulans *and 1549 bp for *Bacillus subtilis*. The size of the 23S rRNAs is in the range 2876 bp for *A. nidulans *to 3122 bp for *Mycobacterium leprae*. The 5S rRNAs are quite small, with an average size of 120 bp and a range extending from 107 bp in *Mycoplasma capricolum *to 123 bp in *Deinococcus radiodurans*. In summary, the average value for n_nuclR _is 4632 nucleotides.

• F_rRNA_. It is believed that the rRNA fraction of total RNA is independent of the growth rate. Specifically, it was shown that in *E. coli*, synthesis of rRNA and tRNA are controlled by similar promoters, so that rRNA and tRNA represent 86% and 14%, respectively, of total stable RNA [25]. Although there are differences between eukaryotic and bacterial ribosomes in RNA sequences and in structure (bacterial ribosomes have two ribosomal RNA molecules, whereas eukaryotes have three), studies of the amount of ribosomal and total RNA in yeast showed that rRNA constituted about 85% of total RNA. This fraction decreased only slightly (to about 83%) with decreasing growth rate [13]. The calculation of rRNA as a fraction of total RNA in the organisms under consideration (Table 3, rows 4 and 5) also shows small variation in this parameter (from 0.82 to 0.86) with an average value of 0.85.

• F_Ra_. A messenger RNA that is being actively translated usually has multiple ribosomes forming a polysome. Translationally inactive mRNAs are often sequestered in messenger ribonucleoprotein particles or associated with a single ribosome. According to experimental observations, the number of ribosomes in the cell is approximately one order of magnitude greater than the number of RNA molecules, but only 10–20% is free and available to initiate translation. The remaining ribosomes are engaged in translation and form polysomes [26]. It is not clear at present how the number of active ribosomes depends on growth conditions and the specific organism. Considering the fact that all other parameters in equation 7 are constants and C is constant, the fraction of active ribosome F_Ra _may not vary significantly among organisms and growth conditions. If we take the number of free ribosomes to equal 15% (between 10 and 20%) then the fraction of active ribosomes will be 0.85.

The C value was calculated by substituting estimated values of the parameters M_nucl_, M_aa_, n_nuclR_, F_rRNA_, F_Ra _in equation 7. The value of C, rounded to the nearest 1000, was 18,000. This number is close to the C value (19,630) obtained by regressing R_RNA:Pr _against R_SGR:PER _(Figure 3). In conclusion, the cellular requirement of ribosomes for protein production in microorganisms may be estimated as the RNA:protein ratio using a general constant to characterise protein-synthesis machinery in the cell. This constant is a function of the number of nucleotides per ribosome, the fraction of ribosomal RNA in total RNA, the fraction of active ribosomes in the cell, and the average mass of nucleotide and amino acid.

Considering the proportionality between R_SGR:PER _and R_RNA:Pr_, the ratios PER:SGR and RNA:protein will be also proportional. Therefore, the overall efficiency of protein production in the microorganisms is proportional to RNA:protein. In conclusion, the effect of growth rate on RNA:protein and P:N ratios actually reproduced and, probably, followed the effect of growth rate on the overall efficiency of protein production in the microorganisms.

## Discussion

### Explaining the similarity of RNA:protein and P:N ratios in diverse organisms

According to equation 6 and Figure 3, the cellular requirement of ribosomes for protein synthesis in both prokaryotes and lower eukaryotes may be calculated from the RNA:protein ratio using the same constant for different unicellular organisms. This emphasizes the similarity in the protein synthesis machinery of different unicellular organisms. Specifically, parameters such as the number of nucleotides per ribosome, the average masses of nucleotides and amino acids, the fraction of ribosomal RNA in total RNA, and the fraction of active ribosomes are similar in unicellular organisms. Conservation of these parameters is consistent with the conservative function of ribosomes. The basic functions of initiation, elongation, and termination in ribosomes of bacteria closely resemble those of eukaryotes. A conservative core of proteins and genes is responsible for the basic "housekeeping" functions of the cell including metabolism, translation, DNA replication and repair, and RNA processing [30,31]. This implies a similarity in genomic mechanisms that regulate growth and protein synthesis of organisms in response to environmental changes and, thus, their RNA:protein and P:N stoichiometry. The actual biomass P:N ratio in organisms is not necessarily equal to the P:N ratio derived from RNA and protein contents. The accumulation of nutrients when there is an excess supply, their reuse, and some other processes may cause significant variation in actual biomass P:N ratios. Averaging the ratios for the organisms among diverse environmental conditions, however, decreases the variation and causes the P:N values to approach cellular growth requirements. It may explain why mean P:N ratios of the microbial species and plants reviewed in the introduction are rather similar to the P: N ratios calculated from RNA and protein contents of the unicellular organism.

Although taking averages of many species reduces N and P variance, the estimation of P:N ratios from RNA:protein for some specific organisms and growth conditions may be invalid. Microorganisms in the studies were considered under conditions of balanced growth or glucose limitation (in the case of *S. ruminantium*). Additional studies are needed to investigate if N and P limitations have a similar effect on the efficiency of protein synthesis, and RNA:protein and P:N ratios. Although the P:N concentrations of many heterotrophic organisms are largely independent of P:N concentrations in the growth media [7], in some microorganisms luxury consumption of nutrients can occur, which could affect the relationships considered in the paper [32]. The relationships for phototrophs and nitrogen-fixing organisms may be even more problematical. Phototrophs have large variations in P:N related to the contents of rubisco and pigments, which vary with light and nutrient supply. In nitrogen-fixing organisms, a large proportion of protein is devoted to nitrogen fixation. Therefore, there is a need for further studies in which both RNA:protein contents and P:N ratios are measured directly and a broader range of organisms and conditions are considered.

### Regulation of the cellular efficiency of protein synthesis, RNA:protein and P:N ratios by trade-off between growth and maintenance

We suggest that the decrease in the cellular requirement of ribosomes for protein production with the increase in cell growth rate reported in this paper may be explained by the disposable soma theory. According to this theory, the metabolic resources are limited in any one organism and may be divided between two main activities, growth/reproduction and maintenance/survival [33,34]. Cellular growth requires a large expenditure of biosynthetic energy in all organisms. Actively growing cells of prokaryotes and low eukaryotes expend around 80% of total nuclear transcription effort in the assembly of translational machinery. About 10% of cell proteins are ribosomal, which are mainly involved in stabilization of rRNAs, thereby facilitating the catalytic roles of ribosomes [35,36]. Thus, microbial cells strictly control ribosome biogenesis by quickly adjusting it to particular nutritional conditions [35,37-39]. This adjustment occurs not only by a decrease or an increase of rRNA transcription, but also by degradation of ribosomes under conditions of bacterial starvation, and by formation of 100S ribosome dimers, which are believed to be a storage form for ribosomes in the stationary state. As stated in the introduction and demonstrated in this study, the mass of total RNA and rRNA in organisms decreases when growth conditions are impaired. Given the disposable soma theory, it may be hypothesized that the reduction of these synthetic efforts coincides with the redirection of metabolic activity in cells from replication to maintenance, and that this redirection is the main reason for a decrease in the RNA:protein and P:N ratios. It is known that, although the activity of "housekeeping" genes involved in ribosome production and other processes related to proliferation decreases in adverse growth conditions, the activity of genes encoding general stress proteins involved in cellular maintenance increases. A long line of evidence supports redirection of this activity in bacteria [40,41].

In conclusion, a stressful environment causes unicellular organisms to conserve cellular resources by directing them into maintenance instead of reproduction. As a result, the decrease in cellular RNA production at slow growth will coincide with a more efficient use of cellular components including ribosomes and synthesized proteins. In combination, these processes decrease the requirement of ribosomes for protein production with decrease in growth rate and, as a result, decrease RNA:protein and P:N ratios.

### RNA:protein ratio as a characteristic of P:N stoichiometry of unicellular organisms and their interaction with the environment

According to the GRH, the main reasons for the positive correlation between P:N stoichiometry of organisms and their growth rate lie in changes in the allocation of cellular resources to the synthesis of ribosomes and ribosomal RNA [6,7]. According to our study, this ecologically important phenomenon may result not only from changes in the production of rRNA, but also from changes in the overall efficiency of protein synthesis. Our findings support the view that, when growth of unicellular organisms is restricted, there is a decrease in the number of ribosomes required to incorporate one amino acid into synthesized protein. These changes decrease RNA:protein ratio in the cell and, therefore, may be responsible for the decrease in P:N stoichiometry of the organism after growth impairment. In support of this mechanism, the P:N ratios calculated from RNA and protein contents in the studied unicellular organisms growing in very diverse conditions were in the same range as the mean ratios reported for different microorganisms, and were similarly related to growth rate as stated in the GRH. The strong links among P:N stoichiometry, RNA:protein ratio, ribosomal requirement for protein synthesis, and growth rate of unicellular organisms indicated by the study have ecological implications. The relationships among the parameters may be used to characterize growth rate and the overall efficiency of protein synthesis of the organism by measuring RNA:protein ratio. In a wider context, it may provide a means of elucidating the N and P economies of complex ecological systems. An example is the N and P economy of soil organic matter. P:N ratios of surface soil organic matter reported in several studies [42-45] are in the same range as the P:N ratio calculated from RNA and protein contents in the studied microorganisms. The other example is the average P:N ratio in oceanic waters, which is close to the P:N ratio in marine phytoplankton [1]. This ratio is equal to the canonical Redfield ratio and also lies in the range indicated by the study. It is plausible, therefore, that the cellular RNA:protein stoichiometry, which characterizes the conservative protein synthesis machinery of the cell, has an impact on the average P:N ratios in different environments.

## Conclusion

Results of the study show that the environment of microorganisms affects their growth rate through trade-off between growth and cellular maintenance. The change in the allocation of the cellular resources in response to growth conditions may affect not only the number of synthesized ribosomes in the cell as postulated by the GRH, but also the overall efficiency of protein synthesis in the cell and, therefore, the RNA:protein and P:N ratios of the microorganisms. This regularity may underlie the similarity in the P:N ratios of diverse organisms. The P:N stoichiometry of organisms, in turn, appears to have a decisive influence on the chemical composition of the natural environment.

## Methods

Experimental data used in the study were obtained from the papers referenced in the Table 1. In all studies except *S. ruminantium*, the organisms were cultured in different growth media supporting their steady state growth at a variety of growth rates. In the case of *S. ruminantium*, a strictly anaerobic ruminal bacterium, the organism was grown in a steady-state continuous culture, under energy (glucose) limitation (as often occurs in the rumen) to control growth rate. No N or P limitation was reported in the studies. The number of growth conditions, temperature, and growth rates of the organisms are given in Table 1. The total weights of proteins were determined in alkaline and hot-acid extracts of cells. The total number of RNA and DNA molecules was estimated in the perchloric acid soluble fraction. In studies of *E. coli, S. coelicolor, M. bovis, S. cerevisiae *(two studies) and *N. crassa*, the relative amount of ribosomal RNA in total RNA was estimated by spectroscopic methods or by separating rRNA on a linear sucrose gradient. The number of active ribosomes in these experiments was estimated as polyribosomes. The distribution of ribosomes in polyribosomes and ribosomal subunits in the studies was determined using zonal sucrose gradient centrifugation. PER were calculated from experimentally determined rRNA contents and the fraction of active ribosomes by equations 1 and 2. The mass of total RNA and proteins per cell were used to calculate (i) the ratio between these parameters by mass (R_RNA:Pr_), (ii) total P and N contents in RNA and proteins per cell, and (iii) the ratio between P and N contents (R_P:N_). The calculations were made for each set of growth conditions in order to estimate their effect on R_RNA:Pr _and R_P:N_. The total P and N contents were not measured in the studies. We calculated these values from the mean weight percentage of these elements in RNA and proteins [5], namely 15% nitrogen and 8% phosphorous in RNA, and 17.2% nitrogen in proteins.

## Abbreviations

**GRH **Growth rate hypothesis

**SGR **Specific growth rates

**SGR**_**max **_Maximum SGR of the organism in the study

**RGR **Relative growth rate (calculated as SGR divided by SGR_max_)

**PER **Peptide chain elongation rate

**R**_**SGR:PER **_Ratio of SGR to PER

**m**_**RNA **_Mass of total RNA

**m**_**Pr **_Mass of proteins

**R**_**RNA:Pr **_Ratio of m_RNA _to m_Pr_

**P **Total mass of phosphorous per cell

**N **Total mass of proteins per cell

**R**_**P:N **_Ratio of P to N by mass

**m**_**rRNA **_Mass of ribosomal RNA per cell

**F**_**Ra **_Fraction of active ribosomes

**n**_**aa **_Cellular protein content in amino acids equivalents

**n**_**R **_Number of ribosomes per cell

**F**_**rRNA **_Fraction of ribosomal RNA in total RNA calculated as the ratio m_rRNA _and m_RNA_

**N**_**a **_Avogadro's constant

**M**_**nucl **_Average mass of nucleotide

**n**_**nuclR **_Number of nucleotides per ribosome

**M**_**aa **_Average mass of amino acid

**C **Coefficient of proportionality between R_RNA:Pran _and R_SGR:PER_

## Authors' contributions

TK developed the concept underlying the study, analyzed data, developed the theoretical model, and drafted the manuscript. DG contributed to analysis and interpretation of the data. CS and TA participated in the acquisition of data and in the critical revision of the manuscript. All authors read and approved the final manuscript.

## Figures and Tables

**Table 1:** Variation of the macromolecular composition, P, and N contents of the unicellular organisms in the studies

**Organism**	**Ref.**	**Number of growth conditions, and temperature (°C)**	**SGR**	**mRNA (fg)**^**c**^	**m**_**Pr**_**(fg)**	**R**_**RNA:Pr**_	**P (fg)**	**N (fg)**	**R**_**P:N**_
*Escherichia coli*	[11]	5 (37)	0.42–1.73	20–212	100–450	0.2–0.47	1.6–16.9	20.2–109.1	0.08–0.15
*Streptomyces coelicolor*	[11]	7 (30)	0.024–0.3	30.9–85.7	144–170	0.21–0.5	2.5–6.9	29.4–42.1	0.08–0.16
*Mycobacterium bovis*	[11]	1 (37)	0.029	13.2	153	0.09	1.1	28.3	0.04
*Selenomonas ruminantium*	[12]	3 (39)	0.05–0.35	53.9–87.2^b^	300–423^2^	0.18–0.21	4.3–7.0^b^	51.6–72.8^b^	0.072–0.081
*Saccharomyces cerevisiae *(1) Strain FL521^d^	[13]	6 (30)	0.04–0.59	600–1400	1900–3040	0.2–0.47	48–112	417–730	0.12–0.15
*Saccharomyces cerevisiae *(2) Strain A364A	[14]	8 (30)	0.085–0.43	490–510	2100–3500	0.15–0.23	39–41	435–680	0.06–0.09
*Neurospora crassa*	[15]	8 (30)	0.09–0.63	15–62.8^a^	132–145^a^	0.11–0.43	5–1.2^a^	25–34.4^a^	0.05–0.15
*Prototheca zopfii*	[16]	12 (25)	0.086–0.223	2200–15610	10000–55000	0.22–0.28	176–1249	2050–11802	0.09–0.13
All organisms			0.029–1.73	13.2–15610	100–55000	0.1–0.5	1.1–1249	20.2–11802	0.04–0.16
Change by a factor of:			60	1183	550	5	1135	584	4

^a^Expressed as w/w.

^b^Expressed as mg per ml.

^c^1 fg = 10^-15^g.

^d^FL521 strain of S.*cerevisiae *carries a mutation and cannot synthesis pyrimidine endogenously, therefore the nucleotides were provided in the growth media.

**Table 2:** Parameters of linear relationships between the P:N ratio in the organisms and their relative growth rate (R_P:N _= a·RGR + b)

**Organism**	**a**	**b**	***R***^**2**^
*Escherichia coli*	0.0573	0.0562	0.99
*Streptomyces coelicolor*	0.2837	0.087	0.85
*Selenomonas ruminantium*	0	0.068	0.09
*Saccharomyces cerevisiae *(study 1)	0.0813	0.1089	0.67
*Saccharomyces cerevisiae *(study 2)	0.0887	0.0523	0.88
*Neurospora crassa*	0.1883	0.0278	0.98
*Prototheca zopfii*	0	0.1	0.02

**Table 3:** Variation in the peptide chain elongation rate (PER), the number of active ribosomes in the cell per one amino acid in the synthesised proteins (R_SGR:PER _)and the fraction of rRNA in total RNA (F_rRNA _)in the studies

**Organism**	**PER, amino acids per second per ribosome**	**R**_**SGR:PER**_**, × 10**^**-5**^**ribosomes per amino acids**	**F**_**rRNA**_	
			
			**Range**	**Mean value**
1	2	3	4	5
*Escherichia coli*	12–21	0.97–2.29	0.851–0.866	0.856
*Streptomyces coelicolor*	0.59–3.17	1.13–2.63	0.851–0.858	0.855
*Mycobacterium bovis*	2.0	0.40	0.875	0.857
*Saccharomyces cerevisiae *(2 studies)	2.8–10.0	0.69–1.20	0.81–0.82	0.82
*Neurospora crassa*	5.35–8.02	0.47–2.18	0.694–0.884	0.857
Range (all the organisms)	0.59–21	0.40–2.63	0.69–0.88	0.82–0.857
Change by a factor of	36	6.5	1.3	1.0
